# Effect of beta-alanine supplementation on the onset of blood lactate accumulation (OBLA) during treadmill running: Pre/post 2 treatment experimental design

**DOI:** 10.1186/1550-2783-7-20

**Published:** 2010-05-19

**Authors:** Thomas Jordan, Judith Lukaszuk, Mark Misic, Josephine Umoren

**Affiliations:** 1 School of Family, Consumer, and Nutrition Sciences. Northern Illinois University, DeKalb, IL, USA; 2 Department of Kinesiology and Physical Education, Northern Illinois University, DeKalb, IL, USA

## Abstract

**Background:**

β-Alanine (βA) has been shown to improve performance during cycling. This study was the first to examine the effects of βA supplementation on the onset of blood lactate accumulation (OBLA) during incremental treadmill running.

**Methods:**

Seventeen recreationally-active men (mean ± SE 24.9 ± 4.7 yrs, 180.6 ± 8.9 cm, 79.25 ± 9.0 kg) participated in this randomized, double-blind, placebo-controlled pre/post test 2-treatment experimental design. Subjects participated in two incremental treadmill tests before and after 28 days of supplementation with either βA (6.0 g·d^-1^)(βA, n = 8) or an equivalent dose of Maltodextrin as the Placebo (PL, n = 9). Heart rate, percent heart rate maximum (%HRmax), %VO_2max_@OBLA (4.0 mmol.L^-1 ^blood lactate concentration) and VO_2max _(L.min^-1^) were determined for each treadmill test. Friedman test was used to determine within group differences; and Mann-Whitney was used to determine between group differences for pre and post values (p < 0.05).

**Results:**

The βA group experienced a significant rightward shift in HR@OBLA beats.min^-1 ^(p < 0.01) pre/post (161.6 ± 19.2 to 173.6 ± 9.9) but remained unchanged in the PL group (166.8 ± 15.8 to 169.6 ± 16.1). The %HRmax@OBLA increased (p < 0.05) pre/post in the βA group (83.0% ± 9.7 to 88.6% ± 3.7) versus no change in the PL group (86.3 ± % 4.8 to 87.9% ± 7.2). The %VO_2max_@OBLA increased (p < 0.05) in the βA group pre/post (69.1 ± 11.0 to 75.6 ± 10.7) but remained unchanged in the PL group (73.3 ± 7.3 to 74.3 ± 7.3). VO_2max _(L.min^-1^) decreased (p < 0.01) in the βA group pre/post (4.57 ± 0.8 to 4.31 ± 0.8) versus no change in the PL group (4.04 ± 0.7 to 4.18 ± 0.8). Body mass kg increased (p < 0.05) in the βA group pre/post (77.9 ± 9.0 to 78.3 ± 9.3) while the PL group was unchanged (80.6 ± 9.1 to 80.4 ± 9.0).

**Conclusions:**

βA supplementation for 28 days enhanced sub-maximal endurance performance by delaying OBLA. However, βA supplemented individuals had a reduced aerobic capacity as evidenced by the decrease in VO_2max _values post supplementation.

## Background

Running is a popular form of exercise in the United States and for many it is considered a competitive sport. While performance goals can range from simply finishing a race to competition in an Olympic event, it is likely that many participants seeking to improve performance use various nutritional supplements.

One such supplement that has recently received interest in improving exercise performance is Beta-Alanine (βA) [1-10]. βA is a non-proteinogenic amino acid that is synthesized in the liver as the final metabolite of uracil and thymine degradation. While produced endogenously, the primary source of βA in humans comes from their diet. Meat is the primary source of dietary βA, with highest concentrations found in chicken and turkey [11]. The performance enhancing potential of βA supplementation lies in its effect on increasing muscle carnosine levels [4,7,8,12] due to its role as the limiting factor in the muscle carnosine synthesis [12-14].

Carnosine (β-alanyl-L-histidine) is a dipeptide found in muscle tissue that acts as an intramuscular buffer of [H^+^] [4,7,8,12]. During high intensity exercise, a greater reliance on the glycolysis and phosphagen systems to supply ATP to working muscles results in an accumulation of [H^+^] which leads to exercise-induced metabolic acidosis [15]. A decline in pH has been implicated as a cause of muscle fatigue and decreased muscle contractile function [16]. Attenuating exercise induced acidosis is purported to result in performance improvements in activities requiring prolonged bouts of high intensity work. This is supported by findings that muscle carnosine concentrations are higher in sprinters [17], bodybuilders [18], and team sport athletes regularly participating in high intensity intermittent exercise [19,20] than in their sedentary counterparts.

Previous studies investigating the effect of βA on performance measures have shown improvements in total work done (TWD) [4,10], time to exhaustion (TTE) [1,4,10], physical working capacity at fatigue threshold (PWCFT) [1,3], power output at lactate threshold (LT) [5], attenuated fatigue during repeated bouts of resistance training [7], and final 30 second sprint performance during a 2 hour time trial [9]. Research has however been conducted using primarily cycle ergometry [1-5,9,10], so it remains to be determined if βA supplementation would have an ergogenic effect during running performance. Therefore, we hypothesized that βA supplementation would delay OBLA. Therefore, the purpose of this study was to determine the effects of 4 weeks of βA supplementation on HR@OBLA, %HRmax@OBLA, %VO_2max_@OBLA, VO_2max _during incremental treadmill running.

## Methods

### Subjects

Seventeen men who were recreationally active and running at least 3 times per week and had not taken any sports supplements for at least 6 weeks volunteered to participate in this study (Table 1). Subjects provided signed consent to participate and all study procedures were approved by the Northern Illinois University Institutional review board prior to enrollment in the study.

**Table 1:** Physical Characteristics of Subjects.

Variable	βA (n = 8)	PL (n = 9)
Age (yr)	24.9 ± 5.1	24.9 ± 4.3
Height (cm)	181.4 ± 9.9	179.8 ± 7.9
Body Mass (kg)	77.9 ± 9.0	80.6 ± 9.1
BMI	23.7 ± 2.3	24.9 ± 1.8

Values are means ± SE

### Experimental Design

This study used a double-blind-placebo-controlled pre/post test 2-treatment experimental design. On days 1 and 29, subjects reported to the exercise lab for anthropometric collection and to perform an incremental treadmill running protocol. During the 28 day study, subjects were randomly assigned to consume a supplement containing either βA (6.0 g·d^-1^) or Placebo (PL) Maltodextrin (6.0 g·d^-1^). Pre- and post-supplementation testing took place at the same time of day for each subject and on the same equipment. Subjects were asked to fast for 2 hrs prior to each test. Subjects were asked to abstain from taking any other dietary supplements and to maintain their regular diet and exercise patterns for the duration of the study. Subjects were also required to abstain from caffeine or vigorous exercise for 24 hrs before exercise testing.

Anthropometric data were recorded in light exercise clothing and bare feet using a wall mounted stadiometer and calibrated digital scale (Tanita Body Composition Analyzer TBF-300A, Tanita Corp, Arlington Heights, IL). Subjects were connected to an automated metabolic measurement system (Parvomedics TrueMax 2400, Consentius Technologies, Sandy, UT) via mouthpiece and headset and fitted with a telemetric heart rate monitor (Polar F6, Finland) in seated position for resting variables prior to testing.

Participants performed 3 minutes of walking on the treadmill at 6.4 km.hr^-1 ^(4.0 mph) to acclimate to the apparatus. The treadmill was then set at a fixed 9.6 km.hr^-1 ^(6.0 mph) for the duration of the test. Every 3 minutes, the treadmill incline was increased by 2% grade. After stage 5, any remaining stages ensued at 3% grade increase (stages: 0%, 2%, 4%, 6%, 8%, 11%, 14%, 17%). The test continued until the participant reached volitional exhaustion. Oxygen uptake was obtained every 30 seconds (s) throughout the test. VO2_max _was recorded as the highest 30 s average recorded prior to volitional exhaustion. Criteria for VO2_max _was attainment of at least two or more of the following: reaching a plateau in VO2 (< 2.1 ml.kg^-1^.min^-1 ^increase) the final two stages of the test, achieving a respiratory exchange ratio (RER ≥ 1.10) and/or reaching a HR within 5 beats per min^-1 ^of predicted maximal value (220 - age). In the final 30 s of each stage, participants were asked to report an overall body rating of perceived exertion (RPE) using a 6-20 numeric scale [21], heart rate was recorded, and a capillary blood lactate sample was collected. Subjects were oriented to the RPE scale prior to initiation of the test.

A fixed marker of 4.0 mmol·L^-1 ^blood was used to define the onset of blood lactate accumulation (OBLA). This fixed lactate measurement provides the most reasonable and accurate lactate analysis relative to the scope of this study and has been shown to be a valid evaluation of physiological changes with specificity to endurance performance [17], and improvements in endurance fitness [18].

Immediately following RPE and HR data collection with 30 s remaining in each stage, subjects while running were asked to extend their left index finger, which was prepared with an alcohol pad and dried with gauze. After preparation, a lancet device was applied to the fingertip and samples were collected in capillary tubes. All lactate samples were immediately analyzed in duplicate using an Accutrend Lactate Analyzer (F. Hoffman-La Roche Ltd, Basel, Switzerland). After compiling the data, the stage that elicited 4.0 mmol/L blood lactate which has been previously identified as the OBLA [22] was used to determine lactate threshold. OBLA, VO2max@OBLA and HR@OBLA were all calculated using linear interpolation between relevant data points as has been previously explained by Neville et al. [23].

The treadmill protocol continued until volitional exhaustion was attained and the highest heart rate experienced during the test was recorded as Max Heart Rate (MHR). OBLA was then also identified by the percentage of maximum heart rate (%MaxHR@OBLA) at which it occurred.

### Supplementation

During the study, subjects were asked to refrain from taking any other dietary supplements or making changes to their regular dietary and exercise patterns. The participants were randomly assigned in a double-blind manner to receive either β-Alanine or Placebo. The supplements were provided to the participants in identical, unmarked, sealed containers, supplied by Athletic Edge Nutrition, Miami, Florida. Subjects received βA supplement (6.0 g·d^-1 ^βA, 600 mg N-Acetylcysteine, 2.7 mg alpha-lipoic acid, 45 IU Vitamin E) or a PL (6.0 g·d^-1 ^Rice Flour Maltodextrin). Both groups followed the same supplementation protocol of 3 capsules 3 times daily with meals.

Supplementing with 6.4 g·d^-1 ^of βA for 28 days has been shown to increase carnosine levels by 60% [4,12] so it can be assumed that supplemented subjects in this study experienced a significant increase in intramuscular carnosine concentration. Three of the eight subjects in the βA supplemented group reported tingling in their fingers and hands. No other side effects were reported by those individuals supplemented with βA and subjects in the PL group reported no side effects.

### Statistical Analysis

Because of the degree of non-normality in the distributions, data transformation could not be done to obtain statistical normality. For this reason, nonparametric statistical methods were used to analyze the data. The Friedman test was used to determine within group differences; and the Mann-Whitney test was used to determine between group differences. Data were analyzed using SPSS for Windows (Version 16.0, 2007 Chicago, IL) Prior to initiation of the study the alpha level was set at p < 0.05 to determine statistical significance. Data are presented as means ± standard error (SE).

## Results

### Participant Characteristics

At baseline there were no differences in age, height, body mass, BMI, absolute VO_2max _L.min^-1 ^(4.57 ± 0.8 βA vs. 4.04 ± 0.7 PL) relative VO_2max _ml.kg.min^-1 ^(58.7 ± 50.0 βA vs. 50.0 ± 5.2 PL) or max HR beats. min^-1^(195 ± 10.2 βA vs. 193.4 ± 14.9 PL) between subjects in the two groups (Table 1). Table 2 presents the mean and standard error values for VO_2max _(L. min^-1^), VO_2max _(ml.kg^-1^.min^-1^), %VO_2max _@ OBLA, VO2@ OBLA (L.min^-1^) MaxHR (beats.min^-1^), HR@OBLA (beats. min^-1^), and %HRMax@OBLA for both treatment groups at pre- and post-testing.

**Table 2:** Exercise Parameters Pre and Post Supplementation.

	Day 1		Day 29	
		
	βA	PL	βA	PL
VO2max (L·min^-1^)	4.57 ± 0.8	4.04 ± 0.7	4.31 ± 0.8**	4.18 ± 0.8
VO2max (ml·kg·min^-1^)	58.7 ± 6.1	50.0 ± 5.2	55.0 ± 6.2**	51.7 ± 5.1
VO2@OBLA (L·min^-1^)	3.16 ± 0.7	2.97 ± 0.6	3.25 ± 0.7	3.11 ± 0.7
%VO2max@OBLA (%)	69.1 ± 11.0	73.3 ± 7.3	75.6 ± 10.7*	74.3 ± 7.3
Max HR (beats·min^-1^)	195.0 ± 10.2	193.4 ± 14.9	196.5 ± 13.1	193.1 ± 9.4
HR @OBLA(beats·min^-1^)	161.6 ± 19.2	166.8 ± 15.8	173.6 ± 9.9*	169.6 ± 16.1
%HRmax @OBLA (%)	83.0 ± 9.7	86.3 ± 4.8	88.6 ± 3.7*	87.9 ± 7.2

Values are means ± SE; *p < 0.05 Pre Supplementation βA vs. Post Supplementation βA **p < 0.01 Pre Supplementation βA vs. Post Supplementation βA

### Absolute (L.min^-1^) and Relative VO2 max (ml.kg^-1^.min^-1^)

On day 1 pre-supplementation there were no significant differences in VO_2max _between subjects in βA and the PL groups (p=.154). On day 29 (post-supplementation) subjects in the βA group had significant decreases in both absolute and relative VO_2max _values (p = 0.005), while no changes were observed in the PL group.

### %VO_2max_@OBLA

On day 1 pre-supplementation there were no significant differences in %VO_2max_@OBLA between subjects in the βA and PL groups. On day 29 (post-supplementation) subjects in the βA group had a significant increase (p = 0.034) in %VO_2max_@OBLA while no changes were observed in the PL group.

### VO_2 _@ OBLA

On day 1 pre-supplementation there were no significant differences in VO_2_@OBLA (L·min^-1^) between subjects in the βA and PL groups. On day 29 (post-supplementation) no changes were observed in the βA group or PL group.

### Heart Rate@OBLA and %HRmax@OBLA

On day 1 pre-supplementation there were no significant differences in heart rate at OBLA (HR@OBLA), or percent maximum heart rate at OBLA (%HRmax@OBLA) between subjects in the two groups. On day 29 (post-supplementation) subjects in the βA group had a significant increase (p = 0.005) in HR@OBLA and %HRmax@OBLA (p = 0.005), while no changes were observed in the PL group. HR @OBLA increased in 8/8 βA supplemented subjects versus 7/9 increased for PL and 2/9 (PL) remained the same post versus pre supplementation. Percent HRmax@OBLA increased in 7/8 (βA) and decreased in 1/8 βA subjects, whereas 6/9 increased for the PL subjects and 3/9 (PL) decreased post versus pre supplementation.

### Body Mass

There was a statistically significant increase in mean body mass for the βA group (p = 0.034) post supplementation while there was no change in the PL group. Mean body mass for the βA group increased by 0.4 kg (77.9 ± 9.0 to 78.3 ± 9.3 kg) following the 28 day supplementation period, while no change occurred in the placebo group (80.6 ± 9.1 to 80.4 ± 9.0 kg).

### Body Mass Index (BMI)

There was a change in BMI values pre/post supplementation (p = 0.034)(βA 23.7 ± 2.3 vs. PL 23.8 ± 2.3) versus post supplementation (βA 24.9 ± 1.8 vs PL 24.8 ± 1.7).

### Rate of Perceived Exertion (RPE)

There were no changes in the final RPE numbers obtained at test termination in the βA group pre/post (18.50 ± .42 to 17.50 ± .82) versus the PL group (18.56 ± .44 to 18.78 ± .32).

## Discussion

While previous studies have suggested an ergogenic effect with βA supplementation in cyclists, this was the first study using running as the exercise protocol. In the current study, results showed that βA supplementation delayed OBLA as illustrated by significant increases in HR@OBLA, %HRmax @ OBLA compared to the PL group. These findings are in part consistent with Zoeller et al. who noted an improvement in power output at lactate threshold on a cycle ergometer [5]. These researchers observed no change in ventilatory measures (VO_2peak _at OBLA), however, it should be noted that Zoeller et al. used a much lower dose of βA (3.2 g·d^-1^) versus the 6.0 g·d^-1 ^used in this study [5].

While muscle levels of carnosine were not measured for this study previous research has indicated that 4-10 weeks of βA supplementation (2.4-6.4 g·d^-1^) increased muscle carnosine levels 37-80% [4,7,8,12] and that a significant relationship exists between carnosine concentration and high intensity exercise performance [19]. Furthermore, carnosine levels are higher in trained athletes [20,24,25] and body builders [26] and have been shown to increase in response to high intensity exercise such as sprint training [27].

### Ergogenic Mechanism of Carnosine

Physically active individuals have higher muscle carnosine concentrations than their sedentary counterparts [20,25-28] and it is clear that both supplementation with βA [4,7,8,12] and high intensity exercise [28] independently increase muscle carnosine levels. While the exact mechanism of action concerning carnosine and exercise performance remains unclear, suggested roles of carnosine include acting as an intramuscular antioxidant [29], regulation of calcium sensitivity and excitation-contraction (E-C) coupling [30,31], protection against glycation by acting as a sacrificial peptide [32], and prevention of protein-protein cross links by reacting with protein-carbonyl groups [33]. The most relevant mechanism of action to this study would be the role of carnosine as an intramuscular buffer against pH decline during exercise.

### Effect of BA Supplementation on Lactate Kinetics

Lactate kinetics following βA supplementation has been evaluated in three previous studies. While lactate is not the cause of the [H^+^] accumulation, the metabolic environment that causes pH decline also increases lactate production, making lactate a good marker for the conditions that induce metabolic acidosis [15]. As suggested by Van Thienen et al., enhanced buffering capacity should allow for enhanced glycolytic energy production and result in an overall increase in lactate production [9]. However, these researchers did not see a significant change in blood lactate concentration following a 30 s cycle sprint at the conclusion of a 110 minute time trial. Derave et al. also failed to show a difference in lactate concentrations 90 and 180 s after a 400 m run for A group as compared with PL group [7]. Conversely, in evaluating lactate concentration during incremental increases in cycling intensity, Zoeller et al. noted an increase in power output (W) at Lactate Threshold [5]. However, the absolute VO_2peak _was unchanged at LT.

### Body Mass

Body mass was increased in the βA group while there was no change in the PL group. This contradicts previous studies reporting no change in body mass in response to βA supplementation [4,8]. Smith et al noted no change in body mass, but did see a significant increase in lean body mass during the first 3 weeks of supplementation (6 g·d^-1 ^βA) in combination with high intensity interval training [10]. Zoeller et al. reported that supplementation with either βA or Creatine alone did not elicit increases in body mass, but in the group receiving both supplements, body mass was increased [5]. Hoffman et al. noted that when subjects were supplemented with either placebo, creatine, βA, or creatine + βA, the creatine + βA group increased lean body mass to the greatest extent [6]. Previous authors have noted that the proposed effects of βA supplementation and an increase lean body mass or body mass is due to a decrease in acidosis along with subsequent increases in training volume [6,10].

### Implications of Study Results

The present study is the first to our knowledge to examine the effects of βA on OBLA during incremental stages of running. After 28 days of 6.0 g·d^-1 ^of βA supplementation, the βA group had a delay in OBLA as determined by increases in HR@OBLA and %MaxHR@OBLA. The findings of this study are consistent with previously discussed studies showing a delay in fatigue after βA supplementation [1-5,7,9,10]. A delay or rightward shift in OBLA during a high intensity exercise offers a significant advantage to an athlete trying to maintain repeated or prolonged high intensity muscle contractions. In addition to the HR findings, there was also an observed increase in %VO_2max_@OBLA within the βA group. However, the authors feel this may be misleading as there was also a decrease in the VO_2max _values post supplementation within the βA group. Therefore, the increase in %VO_2max_@OBLA may simply be due to a decrease in the VO_2max _value. This decrease in VO_2max _was an unexpected finding as it is indicative of a reduced aerobic capacity and is not a typical training response.

### Limitations

The supplement used in this study contained additional antioxidants (600 mg N-Acetylcysteine, 2.7 mg alpha-lipoic acid, and 45 IU Vitamin E) which, per manufacturer claims, may work synergistically with the role of carnosine as an intramuscular antioxidant. However previous research on antioxidants and exercise suggest that an applicable performance enhancement due to antioxidant activity is unlikely [34-40]. Furthermore, the previously suggested ergogenic mechanism of carnosine lies not in its antioxidant function, but in its involvement as an intramuscular buffer [19].

Subjects were asked to not change their regular dietary or exercise habits during the 28 days of the study, refrain from taking any other dietary supplements, avoid caffeine or vigorous exercise for at least 24 hrs prior to exercise testing, and consume 3 pills 3 times daily at meals. Verification of these controls were limited to verbal confirmations by the subjects. Therefore, it may be possible that individuals receiving the βA supplementation were exercising at a greater intensity and this allowed for the significant increase in body mass. Furthermore, although a Tanita scale was used to weigh subjects, body composition data was not collected. Hence, the increase in body mass noted in this study cannot be further differentiated into lean body mass or fat mass.

## Conclusions

The results of this study suggest 28 days of βA supplementation may enhance submaximal endurance performance as measured by OBLA. The authors suggest that βA supplementation may have optimized the relative contribution of the anaerobic energy system but may have also reduced the capacity of the aerobic energy system. More specifically, OBLA was delayed based on higher HR@OBLA and %HRmax@OBLA in the group of individuals receiving the βA versus the PL. Future research is needed to confirm these results and to test for performance related outcomes specific to distance running.

## Future Research

Future studies should focus on looking at the effects of βA on 10-20 km simulated endurance road race performance. With this, a close examination of VO_2max _should be considered. It would also be of interest to determine the ergogenic effects of βA on intermittent sports, such as soccer, hockey, basketball or football, which require a combination of endurance and sprint performance.

## Competing interests

The authors declare that they have no competing interests.

## Authors' contributions

TJ was the primary author of the manuscript and played an important role in the data collection and assessment. JL, MM and JU played an important role in data collection and manuscript preparation. All authors have read and approved the final manuscript.
